# Immunogenicity and safety of a tetanus-diphtheria vaccine and a 13-valent pneumococcal conjugate vaccine after concomitant vaccination in ≥ 50-year-old adults

**DOI:** 10.1186/s12879-018-3479-9

**Published:** 2018-12-05

**Authors:** Joon Young Song, Hee Jin Cheong, Ji Yun Noh, Min Joo Choi, Jin Gu Yoon, Saem Na Lee, Seong Hui Kang, Eun Joo Jeong, Yu Mi Jo, Woo Joo Kim

**Affiliations:** 1 Division of Infectious Diseases, Department of Internal Medicine, Korea University College of Medicine, Seoul, Republic of Korea; 2 Asian Pacific Influenza Institute (APII), Korea University College of Medicine, Seoul, Republic of Korea; 3 Division of Infectious Diseases, Department of Internal Medicine, Konyang University Hospital, Daejeon, Korea; 4 Department of Internal Medicine, Hallym University School of Medicine, Seoul, Korea; 5 Department of Internal Medicine, Gachon University of Gil Medical Center, Incheon, Korea; 6 Division of Infectious Disease, Department of Internal Medicine, Korea University Guro Hospital, 148 Gurodong-ro, Guro-gu, Seoul, 08308 Korea

**Keywords:** Pneumococcal conjugate vaccine, Tetanus, Diphtheria, Immunogenicity

## Abstract

**Background:**

When two or more vaccines are administered concurrently, there is concern about safety and immunogenicity from vaccine interaction.

**Methods:**

Subjects aged ≥50 years were randomized 1:1:1 to receive tetanus-diphtheria (Td) + 13-valent pneumococcal conjugate vaccine (PCV13; Group 1), PCV13 alone (Group 2), or Td alone (Group 3). After single or concomitant vaccination, enzyme-linked immunosorbent assay and opsonophagocytic assay (OPA) were performed to compare immunogenicity for Td and PCV13, respectively.

**Results:**

A total of 448 subjects were available for the assessment. After concomitant administration, the non-inferiority criteria of geometric mean titer (GMT) ratios were met for tetanus, diphtheria, and all four pneumococcal serotypes (1, 5, 18C, and 19A). However, subjects in Group 3 (Td alone) were more likely to have a high IgG anti-tetanus antibody titer (≥ 0.5 U/mL) than those in Group 1 (Td + PCV13) (*p* <  0.01). As for the pneumococcal serotype 1, the OPA GMT was significantly higher in Group 1 (PCV13 + Td) compared to Group 2 (PCV13 alone) (*p* = 0.02). No serious adverse event occurred.

**Conclusions:**

Concomitant Td and PCV13 administration induced sufficient immunity without significant interference and showed good safety profiles.

**Trials registration:**

NCT03552445 registered at http://www.clinicaltrials.gov on June 11, 2018 (retrospectively registered).

## Background

Vaccination is the most effective strategy to prevent diverse infectious diseases. Actually, the World Health Organization (WHO) estimates that vaccinations avert 2–3 million deaths per year [1]. In adults, several vaccines are recommended based on age if the vaccine has not been received before, and there is a lack of evidence of past infection: influenza; measles, mumps and rubella (MMR); varicella; human papillomavirus (HPV); tetanus-diphtheria (Td); tetanus, diphtheria and acellular pertussis (Tdap); and pneumococcal vaccination. In addition, adults should be vaccinated with a variety of vaccines, including those for hepatitis A virus (HAV), hepatitis B virus (HBV), *Haemophilus influenzae* type B (Hib) and meningococcal, based on underlying medical conditions. Thus, adults frequently visit outpatient clinics to receive two or more kinds of vaccines at the same time, as multiple vaccines are given concomitantly during routine pediatric immunizations.

Actually when a patient visits a vaccination clinic, Td and the pneumococcal vaccines are commonly administered at the same time. Pneumococcal vaccines are recommended for chronically ill patients and the elderly aged ≥65 years, while a booster dose of the Td vaccine is required every 10 years from the age of 11–12 years due to waning immunity [2, 3]. Tetanus can be prevented only by vaccination because immunity against this disease is not naturally acquired [4, 5]. Herd protection cannot be induced because tetanus is not person-to-person transmitted.

The development of polysaccharide-protein conjugate technology markedly improved vaccine immunogenicity and enabled the efficient prevention of diverse fatal infectious diseases by encapsulated pathogens such as Hib, *Neisseria meningitidis*, and *Streptococcus pneumoniae*. However, there are concerns about immune interference when multivalent conjugate vaccines are co-administered with other vaccines [6]. There are many kinds of carrier proteins: tetanus toxoid (TT), diphtheria toxoid (DT), CRM_197_ (non-toxic variant of DT), OMP (complex outer-membrane protein mixture from *N. meningitidis*), and non-typeable *H. influenzae*-derived protein D. Depending on the type of carrier proteins and co-administered antigen doses, the degree of immune interference may vary.

In this study, we aimed to evaluate the immunogenicity and safety of the Td vaccine and 13-valent pneumococcal conjugate vaccine (PCV13) after concomitant administration in adults aged 50 years and older.

## Methods

This study is reported according to CONSORT (Consolidated Standards of Reporting Trials) guidelines.

### Study design

This single-center, open-label randomized trial was conducted (Clinical Trial Number - NCT03552445) at Korea University Guro Hospital from November 2013 to April 2016. This study was retrospectively registered at http://www.clinicaltrials.gov on June 11, 2018. Adults aged ≥50 years were randomized in a 1:1:1 ratio to receive Td + PCV13 (Group 1), PCV13 alone (Group 2), or Td alone (Group 3). The block randomization method was used. The vaccines were prepared and injected at the study site by staff members who were not blinded to group assignments; the participants and all other investigators remained blinded to group assignments throughout the trial.

The primary immunogenicity objective of the study was to demonstrate that immune responses to Td antigens one month after vaccination in Group 1 (concomitant administration) were not inferior to those in Group 3 (Td alone). Secondary immunogenicity objectives were to demonstrate that the immune responses to PCV13 serotypes in Group 1 were not inferior to those in Group 2 (PCV13 alone) one month after vaccination. The safety profile of Td + PCV13 compared with that of each agent alone was also assessed.

Healthy adults aged ≥50 years with stable underlying diseases (≥ 6 weeks) were eligible for this study. The exclusion criteria were as follows: 1) a history of pneumococcal infection within the recent five years, 2) previous pneumococcal vaccination, 3) previous Td vaccination within the last 10 years, 4) known immunodeficiency or immunosuppressant use, and 5) coagulation disorders.

The study was approved by the ethics committee of Korea University Guro Hospital (IRB No. 2013GR0005) and was conducted in accordance with the Declaration of Helsinki and Good Clinical Practice. All participants provided written informed consent before enrollment. Venous blood samples of 10 mL were collected on day 0 and post-vaccination day 28 ± 7.

### Vaccines

A 0.5 mL dose of the Td vaccine (SK Chemical Td-pur®, Seoul, Korea), containing 1.5 limes flocculation unit (Lf) diphtheria toxoid and 5 Lf tetanus toxoid with 1.5 mg aluminum hydroxide, was administered intramuscularly into the deltoid muscle.

The PCV13 (Prevnar-13®) vaccine contains polysaccharides from pneumococcal serotypes 1, 3, 4, 5, 6A, 6B, 7F, 9 V, 14, 18C, 19A, 19F, and 23F individually conjugated to nontoxic diphtheria toxin cross-reactive material 197 (CRM_197_). The vaccine is formulated at pH 5.8 with 5 mM succinate buffer, 0.85% sodium chloride, and 0.02% polysorbate 80 and is formulated to contain 2.2 μg of each saccharide, except for 4.4 μg of 6B per 0.5-mL dose. The vaccine also contains 0.125 mg aluminum as aluminum phosphate per 0.5 mL dose. A single dose of PCV13 (0.5 mL) was administered intramuscularly into the deltoid muscle of each participant.

### Immunogenicity assessment

Two different kinds of enzyme-linked immunosorbent assay (ELISA) kits (kit number RE56901 for tetanus and RE56191 for diphtheria; IBL, Hamburg, Germany) were used to determine the serum levels of IgG antibodies to tetanus and diphtheria, according to the manufacturers’ instructions. Antibody levels ≥0.1 IU/mL were considered indicative of seroprotection against their corresponding pathogens [7, 8].

As for the immunogenicity of PCV13, the opsonophagocytic activity (OPA) of the samples was assessed using the validated multiplex opsonophagocytic killing assay (MOPA) as previously described [9]. Target strains SPEC1, STREP5, OREP18C, and TREP19A (expressing capsule types 1, 5, 18C, and 19A, respectively) were derived from wild-type strains L82006, DBL5, GP116, and DS3519–97, respectively, and have been described previously [10]. Each of them was resistant to only one of four antibiotics (spectinomycin, streptomycin, optochin, and trimethoprim). The OPA titer was defined as the serum dilution that kills 50% of bacteria and was determined by linear interpolation. In this study, all sera were diluted five-fold due to the limited sample volumes; hence, the limit of detection was a titer of 20. A detailed protocol is posted online at http://www.vaccine.uab.edu. For MOPA and Td ELISA, laboratory personnel remained blinded at all times.

### Safety assessment

After vaccination, solicited local and systemic reactions were monitored using diary cards during the 14 days post-vaccination. Each subject was asked to record pain, tenderness, and redness diameter at both injection sites and systemic symptoms such as headache, fatigue, chills, myalgia, and arthralgia. Severity was recorded according to the Food and Drug Administration’s Toxicity Grading Scale for Healthy Adult and Adolescent Volunteers Enrolled in Preventive Vaccine Clinical Trials [11]. Any serious adverse events were monitored during the 28 days after vaccination.

### Statistical analysis

Assuming an immune response rate (protective tetanus titers) of 85%, it was projected that 146 subjects per evaluable group would provide at least 80% power to declare a non-inferior tetanus immune response in Group 1 (concomitant administration) compared to Group 3 (Td alone) in older adults ≥50 years of age. Considering a dropout rate of approximately 5% in each group, 462 subjects (154 subjects per group) were planned to be enrolled.

All statistical analyses were performed using SPSS 18.0. Descriptive statistics were reported as numbers and percentages of participants. Tetanus/diphtheria antibody titers and OIs were expressed as geometric means with 95% confidence intervals (CIs). Student’s *t*-tests were used to assess the variation of GMTs between two groups at each time point, and Chi-square tests (Fisher’s exact test was used for < 30 samples) were conducted to compare categorical variables. Statistical significance was defined as *p* <  0.05.

For GMT ratios, CIs were computed using Student’s *t*-tests for the mean difference of the measures on the log scale. Non-inferiority was defined as being met if the lower limit of the two-sided 95% CI for the GMT ratio ([Td + PCV13]/PCV13 or [Td + PCV13]/Td) at one month post-vaccination was > 0.5 (two-fold criterion). Immunogenicity was considered significantly lower if the upper limit of the 95% CI for the GMT ratio was < 1.0.

## Results

### Baseline characteristics

**Fig. 1:**
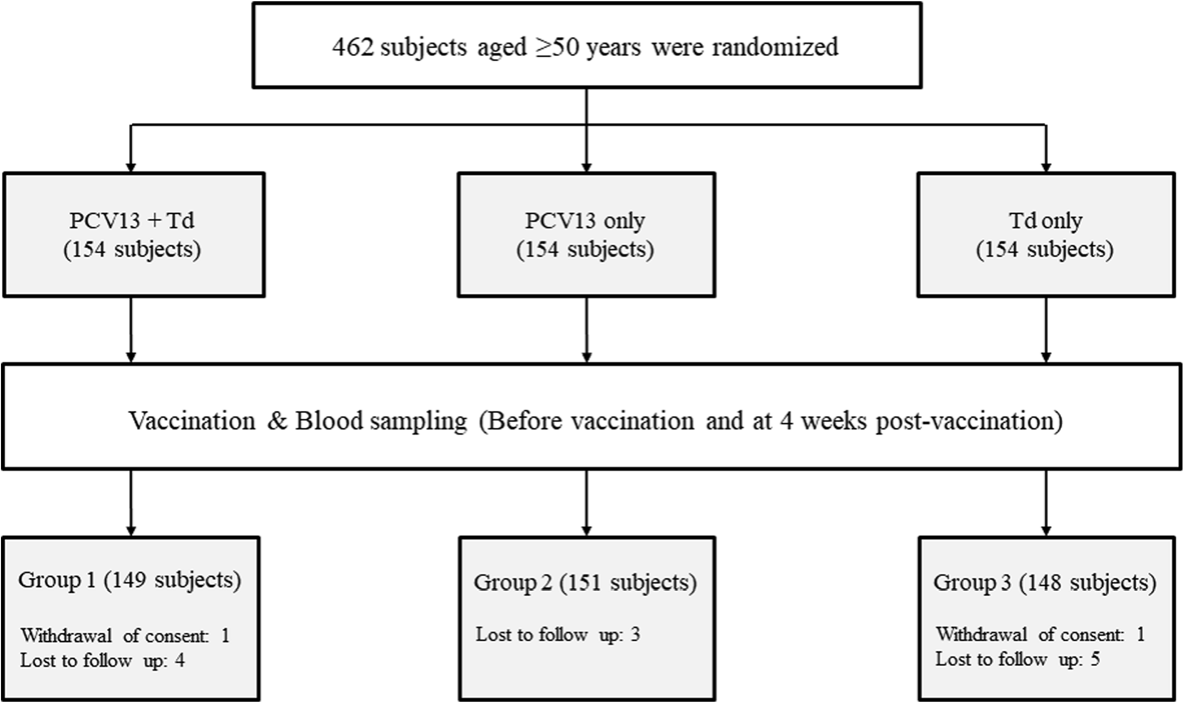
Study flow chart (Td, tetanus-diphtheria; PCV13, 13-valent pneumococcal conjugate vaccine)

A total of 487 subjects were initially recruited, and 462 of them were randomly assigned at a 1:1:1 ratio to one of three vaccination groups: Td + PCV13 (Group 1), PCV13 alone (Group 2), and Td alone (Group 3; Fig. 1). Among the 462 enrolled subjects, 448 (Group 1, *N* = 149; Group 2, *N* = 151; Group 3, *N* = 148) completed the study up to 1 month after the vaccination (Fig. 1). They were included in the assessment of immunogenicity and safety. Baseline demographics were indistinguishable between the study groups (Table 1).

**Table 1:** Baseline characteristics of study subjects

Characteristics	Group 1(*N* = 151)	Group 2 (*N* = 149)	Group 3 (*N* = 148)	*p*-value
Age, mean years (95% CI)	57.5 (56.7–58.4)	57.7 (56.9–58.5)	57.8 (56.9–58.6)	0.91
Age group, No. (%)				0.23
50–64 years	139 (92.1)	139 (93.3)	130 (87.8)	
≥ 65 years	12 (7.9)	10 (6.7)	18 (12.2)	
Male, N (%)	41 (27.2)	37 (24.8)	36 (24.3)	0.84

Abbreviation: *CI* Confidence interval

Group 1: PCV13 + tetanus-diphtheria (Td) vaccine administered concomitantly

Group 2: PCV13 administered alone

Group 3: Td vaccine administered alone

### Immunogenicity

#### Response to td vaccine

**Fig. 2:**
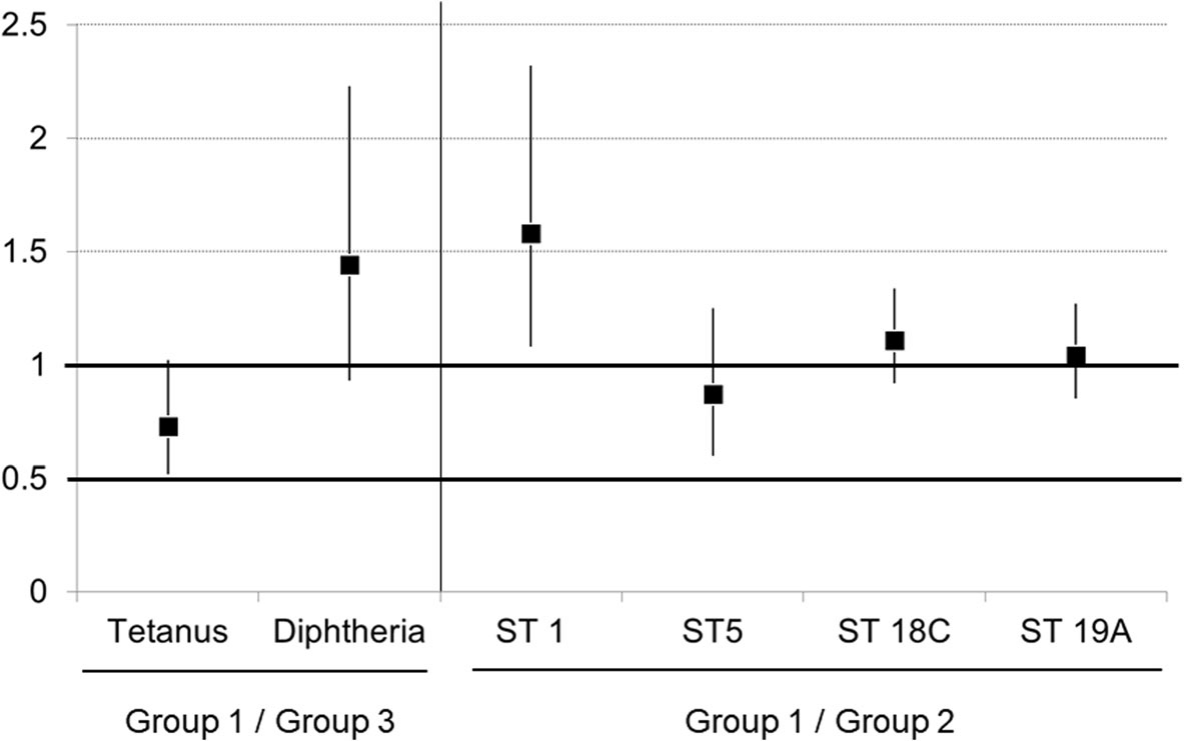
Comparison of geometric mean titers at one month post-vaccination. Enzyme-linked immunosorbent assay: tetanus-diphtheria (Td) vaccine + 13-valent pneumococcal conjugate vaccine (Group 1) versus Td vaccine alone (Group 3). Opsonophagocytic activity (OPA): Td vaccine + 13-valent pneumococcal conjugate vaccine (Group 1) versus 13-valent pneumococcal conjugate vaccine alone (Group 2)

The baseline GMT of the IgG anti-tetanus antibody was rather higher in Group 3 compared to Group 1, but pre-vaccination seroprotection rates of tetanus and diphtheria were similar between the groups (Table 2). After Td vaccination, the seroprotection rates against both tetanus (84.8% versus 87.8%, *p* = 0.50) and diphtheria (79.5% versus 81.1%, *p* = 0.77) were comparable between Groups 1 and 3 at day 28 irrespective of concomitant administration. There was also no significant difference in the GMT fold increase between the two groups: tetanus (8.6 versus 8.5, *p* = 0.47) and diphtheria (13.0 versus 7.9, *p* = 0.14). After concomitant administration, the non-inferiority criteria of the GMT ratio were met for both tetanus and diphtheria (Fig. 2). However, subjects in Group 3 (Td alone) were more likely to have a high IgG anti-tetanus antibody titer (≥ 0.5 U/mL) than those in Group 1 (Td + PCV13) with statistical significance (50.7% versus 26.5%, *p* < 0.01) (Table 2).

**Table 2:** Comparison of immune responses after tetanus-diphtheria (Td) vaccine with or without concomitant 13-valent pneumococcal conjugate vaccine (PCV13)

Antigen	Parameters of immunogenicity	Group 1 (N = 151)	Group 3 (*N* = 148)	*p*-value
Tetanus	GMT fold increase (95% CI)	8.6 (6.6–11.2)	8.5 (6.5–11.2)	0.47
	GMT (95% CI)			
	Pre-vaccination	0.05 (0.04–0.06)	0.07 (0.06–0.08)	0.01
	1 month post-vaccination	0.43 (0.34–0.54)	0.59 (0.46–0.75)	0.06
	Pre-vaccination antibody titer			
	≥ 0.1 U/mL, No. (%)	28 (18.5)	31 (20.9)	0.66
	Post-vaccination antibody titer			
	≥ 0.1 U/mL, No. (%)	128 (84.8)	130 (87.8)	0.50
	≥ 0.5 U/mL, No. (%)	40 (26.5)	75 (50.7)	< 0.01
Diphtheria	GMT fold increase (95% CI)	13.0 (9.1–18.5)	7.9 (5.4–11.7)	0.14
	GMT (95% CI)			
	Pre-vaccination	0.07 (0.07–0.08)	0.08 (0.07–0.10)	0.36
	1 month post-vaccination	0.96 (0.70–1.34)	0.67 (0.50–0.90)	0.10
	Pre-vaccination antibody titer			
	≥ 0.1 U/mL, No. (%)	54 (35.8)	48 (32.4)	0.71
	Post-vaccination antibody titer			
	≥ 0.1 U/mL, No. (%)	120 (79.5)	120 (81.1)	0.77
	≥ 0.5 U/mL, No. (%)	96 (63.6)	91 (61.5)	0.72

Abbreviations: *CI* Confidence interval, *GMT* Geometric mean titer

Group 1: PCV13 + Td vaccine administered concomitantly

Group 3: Td administered alone

#### Response to PCV13

The baseline OPA GMTs of all four serotypes (1, 5, 18C and 19A) were indistinguishable between Group 1 (Td + PCV13) and Group 2 (PCV13 alone; Table 3). For each pneumococcal serotype, OPA titers increased markedly after the PCV13 vaccination, irrespective of the concomitant Td vaccination; all subjects showed an OPA titer ≥8 for serotypes 1, 5, 18C, and 19A post-vaccination. After concomitant administration, the non-inferiority criteria of the GMT ratios were met for all four tested pneumococcal serotypes (Fig. 2). Overall, post-vaccination OPA GMTs were comparable between the two groups. However, in the case of pneumococcal serotype 1, the OPA GMT was significantly higher in Group 1 (PCV13 + Td) compared to Group 2 (PCV13 alone) (*p* = 0.02).

**Table 3:** Comparison of geometric mean titers for opsonophagocytic activity (OPA) after 13-valent pneumococcal conjugate vaccine (PCV13) administration: PCV13 + tetanus-diphtheria (Td) versus PCV13 alone

Serotype	Group	Pre-vaccination OPA		*p*-value	Post-vaccination OPA		*p*-value
		GMT	95% CI		GMT	95% CI	
1	1	11	8–14	0.44	619	465–828	0.02
	2	10	7–12		392	304–506	
5	1	7	6–9	0.09	1140	877–1479	0.45
	2	9	7–11		1312	1016–1690	
18C	1	175	118–259	0.18	10,069	8933–11,350	0.28
	2	248	176–350		9078	7852–10,520	
19A	1	417	303–574	0.40	1517	1349–1706	0.72
	2	346	257–467		1866	1675–2075	

Abbreviations: *CI* Confidence interval, *GMT* Geometric mean titer

Group 1: PCV13 + Td vaccine administered concomitantly

Group 2: PCV13 administered alone

### Safety

Table 4 shows local adverse events within the 14 days after vaccination. There was no significant difference in local reaction (pain, tenderness, redness, and swelling) occurrence among the three groups irrespective of concomitant administration. The most common local reaction was pain at the injection site, which was usually accompanied by tenderness: Group 1 (Td + PCV13, 63.6%), Group 2 (PCV13 only, 56.4%), and Group 3 (Td only, 54.1%). As for the systemic adverse events, the majority of the events were mild in severity (Table 5). Common systemic adverse events were headache (8.8–19.2%), fatigue (14.9–31.31%), chills (7.1–15.4%), myalgia (24.3–38.4%), and arthralgia (4.7–15.9%). PCV13 recipients (Groups 1 and 2) complained of fatigue, myalgia, and arthralgia more frequently compared to Td recipients (Group 3). No serious vaccine-related adverse event was reported.

**Table 4:** Solicited local adverse events within 14 days after vaccination

Local reactions, No. (%)	Group 1 (*N* = 151)	Group 2 (*N* = 149)	Group 3 (*N* = 148)	*p*-value
Pain				
None	55 (36.4)	65 (43.6)	68 (45.9)	0.20
Mild	84 (55.6)	73 (49.0)	76 (51.4)	
Moderate	12 (7.9)	10 (6.7)	3 (2.0)	
Severe	0 (0)	1 (0.7)	1 (0.7)	
Tenderness				
None	55 (36.4)	54 (36.2)	59 (39.9)	0.09
Mild	85 (56.3)	82 (55.0)	86 (58.1)	
Moderate	11 (7.3)	10 (6.7)	3 (2.0)	
Severe	0 (0)	3 (2.0)	0 (0)	
Redness diameter				
0 mm	99 (65.6)	108 (72.5)	117 (79.0)	0.12
1–9 mm	12 (7.9)	9 (6.0)	9 (6.1)	
≥ 10 mm	40 (26.5)	32 (21.5)	22 (14.9)	
Swelling diameter				
0 mm	111 (73.5)	114 (76.5)	123 (83.1)	0.32
1–9 mm	14 (9.3)	15 (10.1)	9 (6.1)	
≥ 10 mm	26 (17.2)	20 (13.4)	16 (10.8)	

Group 1: Tetanus-diphtheria (Td) vaccine + PCV13 administered concomitantly

Group 2: PCV13 administered alone

Group 3: Td vaccine administered alone

**Table 5:** Solicited systemic adverse events within 14 days after vaccination

Systemic reactions, No. (%)	Group 1 (N = 151)	Group 2 (N = 149)	Group 3 (N = 148)	*p*-value
Fever, temp (≥ 38 °C)	1 (0.7)	3 (2.0)	1 (0.7)	0.44
Headache				0.13
None	122 (80.8)	124 (83.2)	135 (91.2)	
Mild	23 (15.2)	22 (14.8)	10 (6.8)	
Moderate	5 (3.3)	2 (1.3)	3 (2.0)	
Severe	0 (0)	1 (0.7)	0 (0)	
Fatigue^a^				0.02
None	104 (68.9)	111 (74.5)	126 (85.1)	
Mild	32 (21.2)	28 (18.8)	19 (12.8)	
Moderate	15 (9.9)	9 (6.0)	3 (2.0)	
Severe	0 (0)	1 (0.7)	0 (0)	
Chills				0.09
None	128 (84.8)	126 (84.6)	139 (93.9)	
Mild	15 (9.9)	14 (9.4)	8 (5.4)	
Moderate	8 (5.3)	8 (5.4)	1 (0.7)	
Severe	0 (0)	1 (0.7)	0 (0)	
Myalgia				0.06
None	93 (61.6)	100 (67.1)	112 (75.7)	
Mild	40 (26.5)	35 (23.5)	31 (20.9)	
Moderate	16 (10.6)	12 (8.1)	3 (2.0)	
Severe	1 (0.7)	2 (1.3)	2 (1.4)	
Arthralgia^b^				0.03
None	127 (84.1)	130 (87.2)	141 (95.3)	
Mild	15 (9.9)	13 (8.7)	4 (2.7)	
Moderate	9 (6.0)	6 (4.0)	2 (1.4)	
Severe	0 (0)	0 (0)	1 (0.7)	

^a^Fatigue was more common in Group 1 compared to Groups 2 and 3. Comparing Groups 2 and 3, subjects in Group 2 complained of fatigue more frequently

^b^Arthralgia was significantly more common in Groups 1 and 2 compared to Group 3

Group 1: Tetanus-diphtheria (Td) vaccine + PCV13 administered concomitantly

Group 2: PCV13 administered alone

Group 3: Td vaccine administered alone

## Discussion

It is very common and efficient to administer two different kinds of vaccines simultaneously for a patient when visiting a clinic. Nevertheless, there are some concerns whether it is safe to administer two vaccines at the same time and whether they can induce sufficient immunity for each vaccine antigen. This study shows that concomitant administration of Td and PCV13 is safe and induces non-inferior immune responses to both vaccine antigens compared to each vaccine alone. Although the pre-vaccination anti-tetanus titer was rather higher in Group 3 (Td alone) compared to Group 1 (PCV13 + Td), seroprotection rates were comparable between the two groups at day 0 (pre-vaccination) and day 28 (post-vaccination; Table 2).

However, an interesting finding in the present study was that the Td vaccine alone induced high IgG anti-tetanus antibody titer (≥ 0.5 U/mL) in a greater proportion than when it was given simultaneously with PCV13. As reported previously, bystander interference might decrease the immune response to co-administered vaccine antigens through competition for limited resources within the lymph nodes and induction of regulatory T-cells [6, 12]. Among carrier proteins, CRM_197_ was suggested to trigger regulatory T-cells, thereby decreasing memory B-cell responses [6]. Although less likely to cause carrier-induced epitopic suppression (CIEP) on polysaccharide antigens compared to TT, CRM_197_ is more likely to induce bystander interference [6, 12]. Multi-valent PCVs with ≥15 serotypes are under development, and they may contain higher doses of CRM_197_ and a larger amount of polysaccharide antigens [13, 14]. Thus, these extended serotype-covering multivalent PCVs might be able to decrease the immune response to co-administered Td or Tdap by bystander interference. Further studies are warranted to better clarify the possible immune interference when these new vaccines are introduced.

On the other hand, co-administration of Td and PCV13 elicited substantially high OPA titers for all four pneumococcal serotypes and induced a superior immune response for serotype 1 pneumococci compared to PCV13 alone in this study (Table 3). It has been suggested that CRM_197_ might induce better immune responses when co-administered or primed with DT [6, 12]. In fact, in previous studies, the response against CRM_197_-conjugated Hib was enhanced with co-administration of DT, suggesting immune enhancement by DT-induced T-helper cells [10, 15]. The CRM_197_-induced CIES effects on polysaccharide antigens may be mitigated by co-administered DT. However, this immune-enhancing effect is not consistently reported in other studies, and observed in a single serotype in the present study. Careful interpretation will be necessary and further research is required. In the studies by Tashani et al., sequential or co-administration of Tdap and PCV13 were compared; OPA titers for PCV13 were significantly higher among concomitant Tdap and PCV13 recipients compared to sequential Tdap and PCV13 recipients [16, 17]. They suggested that prior exposure to Tdap might suppress immune responses to PCV13. Thus, either Td or Tdap vaccination should be scheduled concomitantly or later than PCV13 administration.

As for the safety profile, co-administration of Td and PCV13 is safe and well tolerated. Although PCV13 induced more frequent local pain, concomitant administration of Td and PCV13 had no additive effects on adverse events. The incidences of local and systemic adverse events were comparable to those in previous reports [18–21].

There were some limitations in this study. First, this study was limited by the restricted number of pneumococcal antigens that could be tested (4 of 13). Second, insufficient information was available on previous Td vaccination. Although we only included subjects without Td vaccination in recent 10 years, the number of prior Td vaccination might affect the immune responses against Td antigens.

## Conclusions

When two or more vaccines are administered concurrently, the main concern regarding vaccine interaction is the safety and clinical relevance for individual protection. In this study, the Td vaccine and PCV13 were safe and immunogenic without significant immune interference when administered concomitantly.

## Abbreviations

CIEP: Carrier-induced epitopic suppression
DT: Diphtheria toxoid
ELISA: Enzyme-linked immunosorbent assay
GMT: Geometric mean titer
HAV: Hepatitis A virus
HBV: Hepatitis B virus
Hib: *Haemophilus influenzae* type B
HPV: Human papillomavirus
MMR: Measles, mumps and rubella
MOPA: Multiplex opsonophagocytic killing assay
OMP: Complex outer-membrane protein mixture from N. meningitides
OPA: Opsonophagocytic activity
PCV13: 13-valent pneumococcal conjugate vaccine
Td: Tetanus-diphtheria
Tdap: Tetanus, diphtheria and acellular pertussis
TT: Tetanus toxoid
WHO: World Health Organization
